# The functional biology of peanut allergens and possible links to their allergenicity

**DOI:** 10.1111/all.13719

**Published:** 2019-02-01

**Authors:** Peggy Ozias‐Akins, Heimo Breiteneder

**Affiliations:** ^1^ Genetic & Genomics and Department of Horticulture Institute of Plant Breeding University of Georgia Tifton Georgia; ^2^ Institute of Pathophysiology and Allergy Research Medical University of Vienna Vienna Austria

**Keywords:** evolutionary biology of peanut, functional biology of peanut allergens, geocarpy, peanut pests and pathogens, toxin hypothesis of allergy

## Abstract

Peanut is one of the most common food triggers of fatal anaphylaxis worldwide although peanut allergy affects only 1%‐2% of the general population. Peanuts are the source of highly potent allergenic proteins. It is emerging that the allergenicity of certain proteins is linked to their biological function. Peanut is an unusual crop in that it flowers aboveground but produces its seed‐containing pods underground. This so‐called geocarpic fruiting habit exposes pods and seeds during their development to soilborne pathogens and pests. Pest damage can also open routes of entry for opportunistic fungi such as *Aspergillus*. Although seed proteins have primary functions in nutrient reservoirs, lipid storage bodies, or the cytoskeleton, they have also evolved to act as part of the plant's defense system to enhance fitness and survival of the species. When interacting with pathogens or pests, these proteins modify and damage cells' membranes, interact with immune receptors, and modulate signaling pathways. Moreover, following exposure, the immune system of predisposed individuals reacts to these proteins with the production of specific IgE. This review explores the evolutionary biology of peanut and its seed proteins and highlights possible links between the proteins' biological function and their allergenicity.

## 1 EVOLUTION OF PEANUT

**Figure 1:**
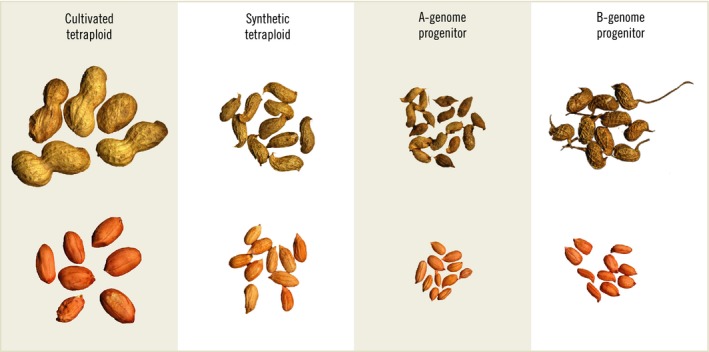
Domesticated tetraploid peanut (*Arachis hypogaea*, AABB genome) was created through spontaneous hybridization and chromosome doubling of the diploid progenitors *Arachis duranensis* (AA genome) and *Arachis ipaensis* (BB genome). Extant accessions of the two species can be artificially hybridized and chromosomes doubled to create synthetic tetraploids that are interfertile with cultivated peanut. Synthetic tetraploids are useful for restoring allelic diversity that was lost due to the domestication bottleneck. Materials and images courtesy of Ye Chu, University of Georgia

Peanut (*Arachis hypogaea* L.) and its wild relatives are endemic to South America having ranged from present‐day Brazil to Bolivia, Argentina, Paraguay, and Uruguay.^1^ Today's cultivated peanut, which is tetraploid (AABB genome), is a member of the legume family (Fabaceae) and evolved from a rare hybridization event of two diploid species, *Arachis duranensis* (AA genome) and *Arachis ipaensis* (BB genome) (Figure 1). Although these two species initially did not occur in the same geographical areas, *A. ipaensis* was almost certainly transported to the center of *A. duranensis* diversity by humans, bringing the two species into close contact.^2^ While cross‐compatible and capable of forming diploid hybrids both in nature and artificially, the differentiated chromosomes of the two diploid species do not pair regularly at meiosis and thus a diploid hybrid is sterile. Either somatic or gametic spontaneous chromosome doubling resulted in the evolution of tetraploid peanut, an event that can be recapitulated with artificial hybridization and induced chromosome doubling using mitotic spindle inhibitors (Figure 1).^3^ Selective pressure was exerted on the interspecific hybrid by humans for its larger seed size, along with its greater productivity compared with previously cultivated diploid species, leading to domestication and cultivation of this important crop.^1^

## 2 DISTRIBUTION AND IMPORTANCE AS A GLOBAL CROP

Cultivated peanut was transported in the 16th century from its primary and secondary centers of origin in South America to Africa and India by Portuguese explorers and to China and Indonesia by Spanish explorers.^3^ It subsequently migrated to North America with the slave trade. Peanut, more commonly called groundnut in Asia and Africa, is now cultivated across subtropical and tropical regions of the world. Four market types, Runner, Virginia, Spanish, and Valencia, are derived from two different subspecies, *A. hypogaea* ssp. *hypogaea* and *A. hypogaea* ssp. *fastigiata*, and are grown, depending on the end use, for oil, in‐shell, confectionary, or peanut butter consumption. Peanut is the fourth most important oilseed globally ranked behind only soybean, rapeseed, and sunflower in production (Food and Agriculture Organization, www.fao.org/faostat; accessed 11/2018). China is the largest producer and India ranks second, with both countries using the crop largely for its oil. While the United States only grows approximately 6% of the world's peanuts, it ranks fourth in production, with peanut gaining popularity as a crop in the early part of the 20th century due to the research efforts of Dr. George Washington Carver. The United States is the most efficient producer with average yields now exceeding 4000 kg/ha compared with <1000 for Africa. The United States also has standard industry measures, such as sorting and aflatoxin testing, to ensure the quality of the products that reach consumers. Unfortunately, consumers in developing countries may consume a low‐quality product if food is scarce. In the United States by 2016, peanut consumption was >3 kg per capita, more than almonds, pecans, walnuts, and pistachios (https://www.ers.usda.gov/; accessed 11/2018). The nutritional benefit of peanut is increasingly recognized even though more than 1% of the population demonstrates allergic reactions to this food ingredient.^4^

Mature peanut seeds, comprised of approximately 45%‐50% oil and 25% protein,^5^, ^6^ are an excellent source of macronutrients as well as minerals and vitamins, especially B and E vitamins and folate.^7^ This nutrient composition has been particularly attractive for the development of ready‐to‐use therapeutic food (RUTF) to treat acute malnutrition in children.^8^, ^9^ The energy‐dense, lipid‐rich RUTF paste remains stable for more than a year without refrigeration and can be administered in the home rather than during a prolonged and disruptive hospital stay. RUTF is regularly administered in more than 50 countries under the guidance of UNICEF. Thus, while allergenicity is a concern in industrialized countries, peanut's ability to save lives is recognized in developing countries.

## 3 GEOCARPY AND ITS CONSEQUENCES

The flowers of *Arachis* develop aboveground and are primarily self‐fertilized, although bee activity can lead to a low frequency of cross‐fertilization and consequent gene flow.^10^ The ovary, at the base of the flower, will develop into the fruit (“pod”) after fertilization of the egg and central cell by the sperm cells released from the pollen tube. A meristem subtending the ovary causes the gynophore (“peg”) to elongate pushing the ovary underground.^11^ There, the growth of the embryo and ovary results in fruit enlargement and maturation. Geocarpy may have been selected as an adaptation to growing conditions in loose soils of alluvial floodplains that may also undergo extreme drought and dry‐season fires. Long‐range dispersal of geocarpic fruits in nature is most frequently accomplished by water in flowing rivers and streams. Hence, isolation in river basins was one evolutionary force that shaped the genus *Arachis*.^12^ However, subterranean fruit development naturally exposes this reproductive structure and its consumable seed to soil microbiota and pests, both beneficial and detrimental.^13^ Immature pods and seeds are highly susceptible to injury by pests and diseases. Since many allergenic peanut proteins are seed storage proteins with putative defense and resistance functions, their synthesis is regulated by seed development.^14^ This developmental regulation of these proteins would play a role in the response of a seed to pests or pathogens.

## 4 PEANUT PESTS AND PATHOGENS

Peanut is susceptible to both foliar and soilborne pathogens and pests,^15^ but this review describes primarily the soilborne group since these pathogens and pests are most likely to damage peanut seeds. Insect, nematode, fungal, and viral pathogens impact the production and quality of peanuts. Many fungi can affect aboveground as well as belowground plant parts including *Sclerotium rolfsii* (white mold or southern blight), *Sclerotinia minor* (sclerotinia blight), *Pythium* spp., and *Rhizoctonia solani*.^16^ White mold is by far the most serious soilborne pathogen in the southeastern United States, where approximately 70% of the US peanut crop is grown, often causing 6%‐8% reduction in crop value.^17^, ^18^ White mold along with the foliar leaf spot diseases (late leaf spot, *Passalora personata*; early leaf spot, *Nothopassalora personata*) and tomato spotted wilt virus (TSWV), a tospovirus, account for the majority of the 13% loss in crop value each year in the southeastern United States.^18^, ^19^ Among fungal pathogens, *Sclerotium rolfsii* is ubiquitous across peanut native and growing regions given its preference for a warm environment where it thrives under conditions of high moisture (https://wiki.bugwood.org/Main_Page; accessed 11/2018). Extreme white mold infection of the pod can result in a dry brown rot. Less severe symptoms are discoloration of the seed coat probably from oxalic acid secreted by the fungus.^15^ Other fungi causing pod rot in peanut are *Rhizoctonia solani* and *Pythium* spp., both cosmopolitan in geographic distribution thus pathogens which peanut is likely to have encountered during evolution. The USDA‐ARS‐GRIN database catalogs reports of fungal‐host associations (https://nt.ars-grin.gov/fungaldatabases/). Fungal infection of pods and seeds may be aggravated by pest damage such as from *Elasmopalpus lignosellus* (lesser cornstalk borer, LCB), *Pangaeus bilineatus* (burrower bug), or *Meloidogyne arenaria* (root‐knot nematode). This is a particular concern for infection by *Aspergillus flavus* or *Aspergillus parasiticus*, the fungi that produce aflatoxins. Fungal infection, however, is not always indicative of aflatoxin production, since the biosynthetic pathway leading to toxin production is triggered by oxidative stress^20^, ^21^ most frequently resulting from exposure of infected pods to water deficit and high temperatures.^22^

**Figure 2:**
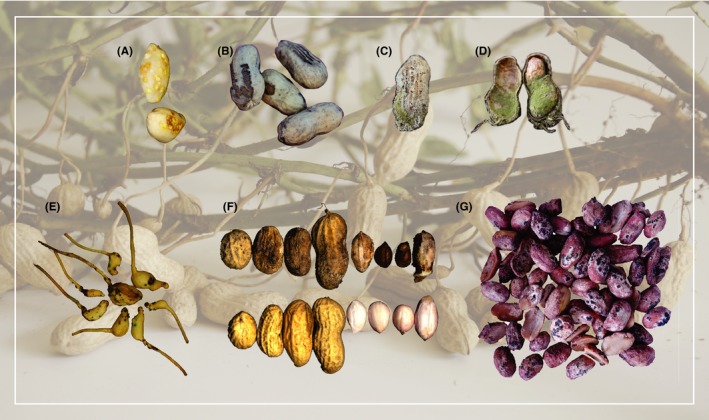
Peanut seeds develop underground and can be exposed to insect, nematode, and fungal pests and pathogens. Of particular concern is damage from (A) burrower bug (*Pangaeus bilineatus*) and (B) lesser cornstalk borer (*Elasmopalpus lignosellus*) both of which are associated with *Aspergillus flavus* colonization (C, D) and may lead to aflatoxin contamination. A similar outcome can result after nematode (*Meloidogyne arenaria*) infection (E) of immature pods shown here as erioglaucine‐stained (blue) egg masses. White mold, one of the more devastating fungal diseases of peanut caused by *Sclerotium rolfsii*, can cause various levels of damage to pods and seeds (F) (top—damaged, bottom—healthy). Sclerotinia blight (*Sclerotinia minor*) may also damage seeds (G). Images were kindly provided by Mark Abney, University of Georgia (A‐D), Larissa Arrais Guimaraes, University of Georgia (E), Kathleen Marasigan, University of Georgia (F), and Rebecca Bennett, USDA‐ARS (G)

The extent of injury to the seed varies with severity of infection or infestation. The more severe manifestations are shown in Figure 2. Nematode injury can slow pod development in part due to root injury but also directly from pod damage. It is likely that nematode damage to the pod also provides a route of entry for *Aspergillus* spp^23^ as does injury due to burrower bug^24^ and lesser cornstalk borer.^25^ The lesser cornstalk borer can either scarify older pods (orange, brown, and black mesocarp)^26^ or penetrate younger pod walls (white or yellow mesocarp)^26^ while the burrower bug enters the seed cavity and directly damages the seed.^24^ LCB's preference for pods in early stages of development results in penetration and access to the seed which can be damaged or consumed. Such young pods contain seeds at an immature stage of development with lower levels of seed storage protein accumulation.^14^

## 5 PEANUT AS AN ALLERGEN SOURCE

**Figure 3:**
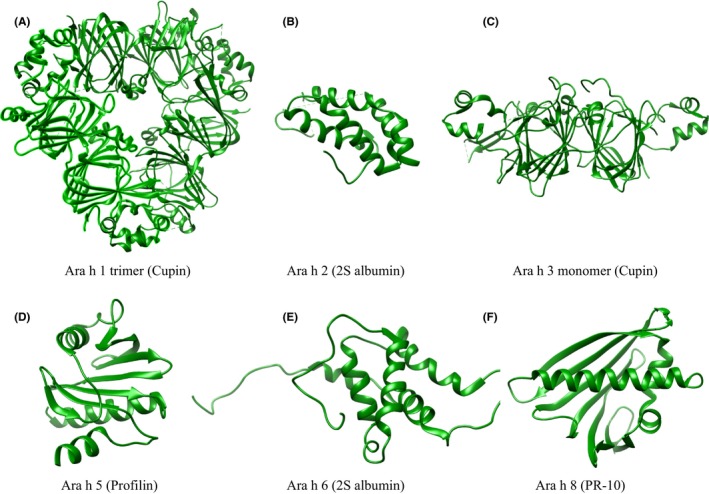
Ribbon representations of the available structures of peanut allergens. A, Ara h 1 (Protein Data Bank accession number 3SMH), (B) Ara h 2 (PDB 3OB4), (C) Ara h 3 (PDB 3C3V), (D) Ara h 5 (PDB 4ESP), (E) Ara h 6 (PDB 1W2Q), (F) Ara h 8 (PDB 4M9B). The images were created with the molecular modeling system UCSF Chimera (https://www.cgl.ucsf.edu/chimera/)

The most common food triggers of fatal anaphylaxis worldwide are peanuts and tree nuts.^27^ Peanuts are the source of an array of highly potent allergenic proteins which can trigger severe anaphylactic reactions even in tiny amounts. At present (11/2018), 16 peanut allergens are officially recognized by the WHO/IUIS Allergen Nomenclature Sub‐Committee (http://www.allergen.org). According to their protein architecture (Figure 3), peanut allergens can be classified into seven groups.^28^ Each of these groups possesses a different degree of allergenic potency.^29^ The USDA reported the per capita peanut consumption in 2016 to be 3.3 kg (https://www.ers.usda.gov/data-products/food-availability-per-capita-data-system/; accessed 11/2018) indicating that a large proportion of the US population is exposed to peanut. Yet, peanut and tree nut allergy in the general US population was reported to affect only 1.4% of adults and 2.1% of children younger than 18 years of age.^4^ Although peanut‐related anaphylaxis is relatively common in peanut allergic individuals, fatalities remain very rare. In general, food allergy‐related fatalities are reported in the range of approximately 0.03‐0.3 deaths per million person‐years in the general US population.^27^

## 6 THE TOXIN AND DAMAGE HYPOTHESES OF ALLERGY

In 1991, evolutionary biologist Margie Profet published the toxin hypothesis of allergy stating that the allergic immune response evolved as a defense mechanism against toxic substances that exist in the environment in the form of secondary plant compounds and venoms.^30^ Experimental confirmation came from the groups of Stephen Galli and Ruslan Medzhitov who showed that IgE directed against bee venom or the allergen Api m 1, a phospholipase A2 from bee venom, could protect mice—once sensitized to sublethal doses—against fatal doses of the toxin.^31^, ^32^ Recent studies have provided evidence that mast cells and IgE play crucial roles in the defense against parasites and arthropod and animal venoms.^33^, ^34^ In 2012, Palm, Rosenstein and Medzhitov published a paper arguing that allergic immunity has an important role in defending the host against venoms, hematophagous fluids, noxious environmental substances, and irritants.^35^ Contrary to the view that allergens are innocuous environmental substances, many of them cause damage to host cells (eg, proteases, defensins) and some are even toxins (eg, phospholipases A2, hyaluronidases). Resistance to pathogens is a prerequisite for the survival of any species. In plants, the seeds require the highest protection. Many proteins of peanut seeds that likely contribute to the defense against pathogens are also allergenic.

## 7 FUNCTIONAL BIOLOGY OF PROTEINS PRESENT IN SEEDS AND PEANUTS

### 7.1 Cupins

The cupin superfamily comprises 65 protein families containing tens of thousands of functionally highly diverse proteins (https://pfam.xfam.org/clan/CL0029; accessed 11/2018).^36^ The term cupin was given to a beta‐barrel domain present in all member proteins of this superfamily. Cupin evolution can be traced from archaea and bacteria to eukaryotes including higher plants.^37^ Bicupins which contain two such beta‐barrels were first identified in the seed storage proteins of higher plants.^38^ The peanut allergens Ara h 1 and Ara h 3 are cupins (Figure 3A,C). Cupins can be divided into the vicilins and the legumins. Vicilins, like Ara h 1, are usually present as 7S trimers. Legumins, like Ara h 3, occur as hexameric complexes. All vicilins of legume seeds are highly heterogeneous and consist of many different subunits. They are the products of multigene families, post‐translational processing, and glycosylation. While the cupin seed storage proteins are a source of amino acids for growth during the germination process, they also possess antimicrobial and insecticidal properties (Table 1).

**Table 1:** Biological functions of proteins with homologues in the peanut seed proteome

Types of proteins (Allergen present in peanuts)	Source	Biological function
*Vicilins* (Ara h 1)	Macadamia nuts	N‐proximal peptides display antimicrobial activity^39^
	Pumpkin, cotton, and cocoa seeds	N‐proximal peptides display activity against plant pathogenic fungi^40^
	Peanuts	N‐proximal peptide of Ara h 1 suppresses the growth of the fungi *Mycosphaerella arachidicola* and *Fusarium oxysporum* 42
	Cowpeas, adzuki beans, common beans, soybeans, and jack beans	Bind strongly to chitin, chitosan, and fully acetylated chitin^43^, ^44^
	Cowpea seed beetle‐resistant cowpeas	Interact with midgut epithelial cells of the cowpea beetle thus interfering with digestive and absorptive processes^46^, ^47^, ^48^
	Cowpeas	Interfere with the germination of spores or conidia of phytopathogenic fungi including *Fusarium oxysporum*.^49^, ^50^
	Soybeans	Inhibit the phytopathogenic fungi *F oxysporum* and *F lateritium* 52
*2S albumins* (Ara h 2, Ara h 6, Ara h 7)	Dandelion seeds	Display inhibitory activity against the mold *Phytophtora infestans* 56
	Passion fruit seeds	Inhibit the growth of the phytopathogenic fungi *F oxysporum* and *Colletotrichum lindemuthianum* 57
	Passion fruit seeds	Permeabilize the plasma membrane of *S cerevisiae* cells^58^
	Sunflower seeds	Interact with lipid vesicles and lipid bilayers^59^
	Sesame seeds	Inhibit *Klebsiella* species^61^
	Castor beans	Disrupt bacterial membranes^62^
*Nonspecific lipid transfer proteins* (Ara h 9, Ara h 16, Ara h 17)	*Arabidopsis thaliana* and spinach seeds	Strongly inhibit bacterial and fungal pathogens^66^
	Onion seeds	Inhibit phytopathogenic fungi^67^ and interact with phospholipid membranes^69^
	Sunflower seeds	Permeabilize intact *Fusarium solani* fungal spores^68^
	Coffee beans	Permeabilize yeast plasma membranes and induce morphological changes^70^
*Oleosins* (Ara h 10, Ara h 11, Ara h 14, Ara h 15)	Plants	Associate with lipid droplets that are enclosed by a monolayer of phospholipids^72^
*Defensins* (Ara h 12, Ara h 13)	Plants	Interact with fungal cell membrane compounds such as sphingolipids or phospholipids^78^, ^79^
	Broad beans	Active against Gram‐positive and Gram‐negative bacteria^81^
	Mung beans	Inhibit alpha‐amylase of larvae of the mealworm *Tenebrio molitor* 82
	Golden rain tree seeds	*Display trypsin inhibitory activity* 83
*Profilins* (Ara h 5)	General	Regulate dynamics of actin polymerization,^85^, ^86^ bind more than 50 ligands including regulators of endocytosis, nuclear export receptors, and small GTPases^88^
	*Toxoplasma gondii*	Bind to and induce signaling through the murine TLR11 and TLR12^90^
*Plant pathogenesis‐related proteins PR‐10* (Ara h 8)	Peanut and cacao seeds	Possess antifungal activity^94^, ^95^
	Oca, an Andean tuber crop	Inhibit the growth of several phytopathogenic bacteria and fungi^96^
	Roots of the legume *Crotalaria pallida*	Inhibit digestive proteinases from the root‐knot nematode *Meloidogyne incognita* 97
	Birch pollen	Bind to and significantly perturb lipid bilayer structure^98^

A vicilin seed storage protein of macadamia nuts contains a 28 amino acid (aa) N‐terminal signal sequence, an N‐proximal extremely hydrophilic region of 212 aa, and a 426 aa C‐terminal region present in all vicilins.^39^ The N‐proximal region comprises four segments of about 50 aa each possessing a C‐XXX‐C‐(10‐12)X‐C‐XXX‐C motif. These four‐cysteine‐type antimicrobial peptides (AMPs) are released when the vicilin of macadamia nuts is processed during seed germination and display antimicrobial activity. Pumpkin, cotton, and cocoa vicilins contain similar segments possessing the four‐cysteine motif. These peptides are important elements in plant defense against plant pathogenic fungi.^40^ The presence of a cysteine‐rich hydrophilic region proximal to the N‐terminal signal peptide is also a feature of Ara h 1 from peanut. The signal peptide directs Ara h 1 to the storage vacuole where this N‐proximal peptide is cleaved off. It contains three IgE‐binding epitopes, two of which are major.^41^ This peptide was later isolated from peanut seeds, named hypogin and shown to suppress the growth of the fungi *Mycosphaerella arachidicola*,* Fusarium oxysporum*, and *Coprinus comatus*.^42^ To date, hypogin has not been included in the list of official peanut allergens.

Vicilin storage proteins isolated from the seeds of cowpea (*Vigna unguiculata*), adzuki bean (*V radiata*), common bean (*Phaseolus vulgaris*), soybean (*Glycine max*), and jack bean (*Canavalia ensiformis*) were shown to bind strongly to chitin, chitosan, and fully acetylated chitin.^43^, ^44^ Cowpeas are the preferred host seeds for the cowpea seed beetle (*Callosobruchus maculatus*) causing severe postharvest losses. Vicilins isolated from a resistant cowpea line strongly inhibited *C. maculatus* larval development.^45^ Vicilins from resistant cowpeas were shown to bind strongly to chitinous structures present on the apical part of the microvilli from the midgut epithelium of *C. maculatus* larvae.^46^ This interferes with digestive and absorptive processes resulting in substantial growth inhibition of larvae fed on resistant seeds. The toxicity of these vicilins seems to be related to their interaction with *N*‐acetylglucosamine containing glycoproteins and other microvillar membrane constituents prior to their internalization by enterocytes which results in interference with the physiology of these cells.^47^ There is evidence that the internalization of vicilins into midgut epithelial cells of *C. maculatus* larvae is mediated by an enterocyte microvillar membrane‐bound receptor with homology to alpha‐tocopherol transfer proteins.^48^

Cowpea vicilins also interfere with the germination of spores or conidia of phytopathogenic fungi including *F. oxysporum* and inhibit yeast growth by binding to various sugars present in fungal cell walls such as *N*‐acetylglucosamine, sucrose/glucose, and glucosamine.^49^, ^50^ As soon as they are rehydrated, germinating cowpea seeds exudate a variety of defense‐related proteins such as vicilins and nonspecific lipid transfer proteins to protect the seeds from pathogens present in the soil.^51^ Vicilins are also present in the seed coat of legumes such as the soybean.^52^ Following rehydration of the seeds, these vicilins together with acid phosphatase and peroxidase were released and shown to inhibit the phytopathogenic fungi *F. oxysporum* and *F. lateritium*.

### 7.2 Prolamins

The prolamin superfamily contains several protein families with only limited sequence identities. The superfamily received its name from one of its member families, the prolamins which are major seed storage proteins in most cereal seeds. Parts of the non‐repetitive domain of one group of the sulfur‐rich cereal prolamins are homologous to sequences present in a large group of low molecular and heavily disulfide‐bonded seed proteins including the 2S albumins, the nonspecific lipid proteins (nsLTPs), and the cereal inhibitors of α‐amylase and trypsin.^53^ The prolamin superfamily seems to be of a much more recent origin than the cupin seed storage proteins. nsLTPs have most likely only evolved after plants have conquered land as they are abundant in land plants but have not been found in any algae.^54^

#### 7.2.1 2S albumins

The peanut allergens Ara h 2, Ara h 6, and Ara h 7 are 2S albumins (Figure 3B,E). 2S albumins in seeds are a source of nutrients during germination but also possess antifungal and antibacterial properties (Table 1). In 1992, Terras and colleagues described for the first time that 2S albumin seed storage proteins were able to inhibit the growth of a large spectrum of fungi.^55^ 2S albumins from seeds of dandelion were shown to possess inhibitory activity against the mold *Phytophtora infestans*.^56^ 2S albumins isolated from the seeds of passion fruit (*Passiflora edulis*) inhibited the growth of the phytopathogenic fungi *F oxysporum* and *Colletotrichum lindemuthianum* and the yeast *S cerevisiae*.^57^ Passion fruit seed 2S albumins were able to permeabilize the plasma membrane of *S cerevisiae* cells leading to the dissipation of the proton gradient across the membrane.^58^ The treatment further resulted in changes in yeast morphology affecting the cell surface, cell wall, bud formation, and the organization of organelles. 2S albumins from sunflower seeds were shown to possess excellent emulsification properties indicating their ability to interact with lipid vesicles and lipid bilayers.^59^ The ability of SFA‐8, a specific sunflower seed 2S albumin, to form highly stable emulsions with oil/water mixtures may be determined partly by a hydrophobic patch on the surface of the protein.^60^ Several 2S albumins have been reported to possess bactericidal activity. A member of the 2S albumin family from sesame seeds (*Sesamum indicum*) specifically inhibited *Klebsiella* species.^61^ A 2S albumin from castor beans (*Ricinus communis*) was reported to have high in vitro antibacterial activity against human pathogenic bacteria.^62^ Atomic force microscopy indicated that this 2S albumin disrupted the bacterial membranes resulting in the loss of cytoplasm and bacterial death.

#### 7.2.2 Nonspecific lipid transfer proteins (nsLTPs)

The peanut allergens Ara h 9, Ara h 16, and Ara h 17 are nsLTPs. The nsLTP family is divided into the 9 kDa nsLTP1 subfamily and the 7 kDa nsLTP2 subfamily.^63^ NsLTP1 is primarily found in aerial organs, while nsLTP2 is expressed in roots. Both nsLTP1 and nsLTP2 are found in seeds. Both types possess an internal cavity comprising potential binding sites for hydrophobic and amphiphilic molecules. NsLTPs are involved in essential cellular processes such as biogenesis and stabilization of membranes, cell wall organization, and intra‐ and intercellular signaling but they also play important roles in resistance to biotic and abiotic stress, plant growth, and development (Table 1).^64^, ^65^ Many nsLTPs display antimicrobial activity and inhibit the growth of pathogenic fungi and bacteria. nsLTPs from *Arabidopsis thaliana* and spinach were shown to be potent inhibitors of bacterial and fungal pathogens.^66^ An nsLTP from onion seeds was reported as a potent antimicrobial protein that inhibited an array of phytopathogenic fungi.^67^ Plant nsLTPs also have fungicidal activity. They are able to permeabilize cell membranes of pathogenic fungi. Liposome leakage assays showed that a sunflower seed nsLTP induced the release of fluorescent probes encapsulated in model membranes, indicating the protein's ability to interact with phospholipids. The sunflower nsLTP was also able to induce the permeabilization of intact *Fusarium solani* fungal spores.^68^ Likewise, an nsLTP from onion seeds was able to interact with phospholipid membranes as shown by the release of carboxyfluorescein from the lumen of artificial liposomes.^69^ An nsLTP from coffee beans with strong antifungal activity against *Candida albicans* was able to permeabilize yeast plasma membranes and induced morphological changes including the formation of pseudohyphae *in Candida tropicalis*.^70^ It was suggested that the lipid‐binding activity of nsLTPs as well as their positive charge which allows the interaction with negatively charged components of biological membranes of phytopathogens results in the destabilization of the membrane structure.^71^

### 7.3 Oleosins

Neutral lipids in plants are stored within cytoplasmic lipid droplets and serve as energy and carbon sources during the growth and development of the seedling. A lipid droplet has a core of neutral lipids enclosed by a monolayer of phospholipids and proteins, which play structural and/or metabolic roles.^72^ The major proteins that specifically associate with these lipid droplets are oleosins, caleosins, and sterol dehydrogenases.^73^ The peanut allergens Ara h 10, Ara h 11, Ara h 14, and Ara h 15 are oleosins. Oleosins bind to the surface of lipid droplets and ensure their structural integrity during seed desiccation and rehydration (Table 1). Oleosins have a polar C‐ and N‐terminus flanking a central hydrophobic hairpin capable of penetrating the phospholipid monolayer and inserting into the hydrophobic core of an oil droplet.^74^ Oleosins evolved in green algae, the predecessors of modern plants.^75^ No studies are available on the effect of plant oleosins on membranes of mammalian cells. It is tempting to speculate that the local accumulation of oleosins on epithelial lipid bilayer membranes may modify their curvature and consequently destabilize the membrane structure. Thus, the lipid‐binding activity of oleosins may be directly involved in membrane destabilization of epithelial cells.

### 7.4 Defensins

Defensins are an extensive group of small, cationic, disulfide‐rich proteins found in animals, plants, and fungi. Defensins are part of an organisms' innate immune system with activities directed against fungi, bacteria, and insects (Table 1).^76^ The peanut allergens Ara h 12 and Ara h 13 are defensins. Plant defensins are characterized by a disulfide‐stabilized alpha‐beta protein fold which resembles the structure of insect and vertebrate defensins.^77^ However, the antimicrobial activity of plant defensins is largely directed against specific fungal lipids, inducing the production of reactive oxygen species or causing cell wall stress. Defensins interact with fungal cell membrane compounds such as sphingolipids or phospholipids.^78^, ^79^ Following binding of their targets, plant defensins can either stay at the cell surface and induce cell death through specific signaling cascades or they can be internalized and interact with intracellular targets.^80^

Antibacterial activity is less common in plant defensins. The fabatins from the broad bean *Vicia faba* were shown to be active against Gram‐positive and Gram‐negative bacteria but inactive against yeasts.^81^ A defensin from mung bean was reported to inhibit the alpha‐amylase of mealworm (*Tenebrio molitor*) larvae.^82^ A defensin isolated from the seeds of the golden rain tree (*Cassia fistula*) displayed trypsin inhibitory activity.^83^ Certain plant defensins inhibit mammalian potassium channels by physically blocking them and show structural similarities to certain sodium and potassium channel blocking scorpion toxins.^84^

### 7.5 Profilins

The profilin family of proteins is one of the four member families of the profilin‐like superfamily. Profilins are small cytoplasmic proteins and are present in all eukaryotic cells. Profilins are involved in regulating the dynamics of actin polymerization (Table 1).^85^, ^86^ The peanut allergen Ara h 5 is a profilin (Figure 3D). Besides the binding site for actin, profilins also possess binding sites for phosphoinositides and for poly‐L‐proline stretches. The first proline‐rich protein identified as a profilin ligand was the vasodilator‐stimulated phosphoprotein.^87^ Since then, more than 50 ligands from different organisms have been identified including regulators of endocytosis, nuclear export receptors, and small GTPases.^88^ As endogenous profilins are involved in complex networks of molecular interactions, external profilins might interfere with their proper functions. Exogenous profilins might gain access into the system in individuals with impaired epithelial barrier integrity.^89^ Exogenous profilins from pathogens are also able to trigger innate immune receptors as was shown for the soluble *Toxoplasma gondii* profilin that binds to and induces signaling through the murine TLR11 and TLR12.^90^

### 7.6 Plant pathogenesis‐related proteins pr‐10

The Bet v 1‐like superfamily of proteins received its name from the major birch pollen allergen Bet v 1. The Bet v 1 architecture evolved at the beginning of cellular life and hence this fold became distributed into all kingdoms of life.^91^ The Bet v 1‐like superfamily consists of 19 families including the Bet v 1 family (https://pfam.xfam.org/clan/CL0209; accessed 11/2018). The Bet v 1 family comprises 11 subfamilies, one of them being the PR‐10 group of proteins (Table 1). Most of the Bet v 1‐homologous allergens known today belong to the PR‐10 subfamily.^92^ The peanut allergen Ara h 8 is a PR‐10 protein (Figure 3F). The PR‐10 fold consists of a seven antiparallel beta strands and three alpha helices enclosing a large hydrophobic cavity which is most probably one of the keys to their biological function.^93^ Several PR‐10 proteins were shown to possess antifungal activity including AhPR‐10 from peanut and TcPR‐10 from cacao.^94^, ^95^ Both proteins were shown to be internalized by fungal hyphae via an active uptake mechanism. Ocatin, a PR‐10 protein from the Andean tuber crop oca (*Oxalis tuberosa*), was revealed to inhibit the growth of several phytopathogenic bacteria and fungi.^96^ CpPRI, a PR‐10 protein purified from roots of the legume *Crotalaria pallida*, was shown to act against a digestive proteinase from the root‐knot nematode *Meloidogyne incognita* and demonstrated nematostatic and nematicidic effects on this parasite in bioassays. Moreover, CpPRI was observed to be internalized and diffused over the entire body of *M. incognita*.^97^ The major birch pollen allergen Bet v 1 was shown to bind to lipid bilayers, undergoing a major structural rearrangement in the process, and to significantly perturb the bilayer structure.^98^

## 8 CONCLUSION

Many potent allergenic proteins are far from being inert and harmless environmental substances. Their biological functions encompass activities that modify and damage cells' membranes, interactions with innate immune receptors, and modulation of signaling pathways (Table 1). The evolution of peanut and its characteristic of pushing the developing pods underground and thereby exposing them to an array of pathogens have favored the development of seed proteins that act as part of a plant's defense system. Interestingly, these proteins are recognized as allergens by the immune system of predisposed individuals. More research is needed to substantiate this proposed and potential link between the biological function and the allergenicity of peanut seed proteins.

## CONFLICTS OF INTEREST

The authors declare that they have no conflicts of interest.
